# 

**Published:** 1844-06

**Authors:** 


*To Correspondents.*—The Baltimore Editor having been absent for several months, on a tour through the Southern and Western States, found, on his return, a large number of letters and communications from correspondents, some of which should have been replied to immediately. His absence from the city, he hopes, will be received as a sufficient apology for what might otherwise be regarded as neglect on his part. They shall receive immediate attention after his return from New York, which place he is compelled to visit at once, to attend the Fifth Annual Meeting of the American Society of Dental Surgeons.

*To our Subscribers.*—The delay in the publication of the present number of the Journal was occasioned by the absence of the Baltimore Editor.

*Gossip with our Profession, by the Junior Editor.*—We perceive, with regret, that some of the members of the American Society of Dental Surgeons, have mistaken the language of the Society's Diploma—supposing it conferred the degree of doctor of dental surgery. Such are informed that the Society has not the power, (being yet unchartered,) to confer *any* degree—its Diploma being a certificate of membership only. The Baltimore College of Dental Surgery is the only institution having authority to confer the degree of D. D. S. . . . If those of our profession who do not take the Boston Medical and Surgical Journal, could know how much of its excellent contents have a direct connexion with our own science, and how cordially the interests of our profession are advocated by its courteous editor, there would be few, we opine, who could hesitate an instant about paying three dollars a year for so valuable a periodical. . . . . Filling teeth with any paste or cement has, in our neighborhood, gone out of practice, we believe; and we heartily congratulate the people thereupon. We have recently had occasion to operate for a gentleman, greatly distinguished for his inventions and productions in mechanics, who had his teeth filled with Mercurial Paste, in London, and, in consequence, lost his health, and nearly lost his life. It is our intention, (if permitted) to report the case for this Journal, and to accompany the report with the testimonials of some of the highest medical authorities in this region. . . . . There is advertised in the "London Times," a cement, patronized by their Majesties, Highnesses, &c.—with which people may plug their own teeth! We expect the next reach in humbuggery will be some new operation of lithotomy, to be performed upon the patient by himself. M.

*Washington City, July,* 1844.

### INDEX.

PAGE.

Address, Opening, by C. A. Harris, M. D., . . . . 3
Address, before the Virginia Society of Dental Surgeons, by J. D. McCabe, D. D. S., . 99
Address, before the Cincinnati Association of Dental Surgeons, by M. Rogers, M. D., 258
Address, Valedictory, by Professor T. E. Bond, . 221
Absorption of the Roots of the Temporary Teeth, &c., translated from the French of Delabarre, by C. A. Harris, M. D., . . . . . 279
Alveolar Exostosis, by S. M. Shepherd, D. D. S., . . 53
Artesian Wells, . . . . 64
Abscess, Dental, . . . . 59
Abscess of the Teeth, . . . 58
American Society of Dental Surgeons, fourth meeting, . 69
Advantage of System in Medical Inquiries, . . . . . 121
Animal Heat, Theory of . 123
Accident to Baron Berzelius, 68
Artificial Teeth, . . . . 72
Apparel the, destined by Nature for the Absorption of the Roots of the Temporary Teeth, . 279

B

Bond, Professor T. E. Jr., on the Morbid Sympathies between the Mouth and other parts of the Body, . . . . 23–74
Obituary Notice of Professor Hayden, being a Valedictory

PAGE.

Address delivered before the Graduate Class, . . . 221
Berzelius, Baron, accident to, 68
Brown, Solyman, M. D., case of Dr. J. S. Walcott. . . 145
Blow-pipe, Dr. R. Somerby's Concentrated, . . . . 218
Burning Wells of Kenawha, the 294

C

Caries, Dental, by A. Wescott, M. D., . . . . 31–73
Caries of the Upper Jaw, Regeneration of the Teeth, . 64
Caries, Dental, . . . . 65
Contributions to Operative and Mechanical Dentistry, by W. H. Elliott, . . . . 45–162
Cure of Abscess of a Tooth, 58
Correspondence, . 73–75–218
Climate and Characteristics of Nice, . . . . . 122
Cataract, Operation for, . 128
Clendon, J. C., on the extraction of Teeth, . . . . 128
Cutlery, . . . . . . 137
Coadjutor, our late, . . 141
Calculus, Salivary, . . . . 141
Cheek and Lip, retractor for the, by James Gunnell, M. D., . 174
Cushman, the Dental Surgeon defined, . . . . . 201
Compliment to the Memory of Sir Chas. Bell, . . . . 214
Cheap Dentist, . . . . 215
Orbital Cavity, Cyst in the, 296
Correspondents, . . . . 300

PAGE.

D

Dissertation on the Morbid Sympathies between the Mouth and other parts of the Body, by Professor T. E. Bond, Jr. 23
Dissertation of Dental Caries, by A. Wescott, M. D., . 31
Dentistry, Mechanical, &c., by W. H. Elliott, . . 45–162
Denture, Irregularity of the Superior, by Edward Maynard, 55
Dental Abscess, . . . 59
Dental Surgery in the U. S. . 67
Dental Surgeons, American Society of, . . . . . 69
Dental Journal, British, . . 72
Dental Ingenuity, . . . 120
Dental Surgeon defined, by C. T. Cushman, . . . 201
Dentist, Cheap, . . . 215
Dental Surgery, Baltimore College of, . . . . . 298

E

Elliott, W. H., Contributions to Operative and Mechanical Dentistry, . . . . 45–162
Exostosis, Alveolar, by S. M. Shepherd, D. D. S., . . 53
English General Practitioner, 67
Extract from a letter to the Baltimore Editor, by Dr. J. C. Mc Cabe, . . . . . . 88
Errata, . . . . . 144
Extracting Forceps, by W. W. H. Thackston, D. D. S., 166
Essay on the Morbid Effects of Diseased Teeth upon the Human System, by W. W. H. Thackston, D. D. S., . 230

F

French Medical Societies, . 126
Forceps, Extracting, by W. W. H. Thackston, D. D. S., . 166
Filling Teeth, Metallic Paste for, 210

G

Gunnell, J. S., M. D., on an affection produced by the Dentition of the Lower Dens Sapientiæ, . . . . 43
Gunnell on the Cheek and Lip Retractor, . . . 174
Gum, Suppression of Hæmorrhage at the, . . . 65
Gums, Ulceration of the, and Exfoliation of the Alveolar Processes, by Prof. C. A. Harris, 93
Gidney, Dr. Eleazar, . . . 142
Gossip with the Profession, by the Junior Editor, . 142–219

H

Harris, C. A., M. D., Opening Address, . . . 3
Harris, C. A., M. D. on Ulceration of the Gums, and Exfoliation of the Alveolar Processes, 93
Hæmorrhage at the Gum, Suppression of, . . . 65
Handy, Professor W. R., Annual Introductory Lecture, 77
Heat, theory of Animal, . 123
Hullihen, Dr. S. P., on Aneurism by Anastomosis of the Superior Maxillare, . . 160
Hayden, H. H., M. D., Obituary of, . . 133–221
Hare-lip and its Treatment, by S. P. Hullihen, M. D., . 244

I

Irregularity of the Superior Denture, by Ed. Maynard, M. D. 55
Illegal Practice of Medicine in Paris, . . . . . 66
Incorruptible Teeth, . . 72
Illustrations and Sketches of Medical Quackery, . 121

J

Journal, British Dental, . 72

L

Lecture, Introductory, by Professor W. R. Handy, M. D., 77
Letter, Extract from a, to the Baltimore Editor, by Dr. J. C. McCabe, . . . . . 88

PAGE.
Leeches, how to make them bite 128
Lithodeon, . . . . . 138

M

Maynard, Edward, M. D., on Irregularity of the Superior Denture, . . . . . 55
Mouth, Morbid Sympathies between the, and other parts of the Body, Professor T. E. Bond, . . . . 23
Morbid Effects of Diseased Teeth &c., &c. by W. W. H. Thackston, D. D. S., . . . 230
Mouth, Tartar or Calculus of the, translated from the French of Delabarre, by C. A. Harris, M. D. . . . . . . 268
Mechanical and Operative Dentistry, Contributions to, by W. H. Elliott, . . . 45–162
Meteorology, Science of, . 63
Medical Things in Paris, . . 67
Medical Societies, French, 126
" Writers, good Practitioners, . . . . . 206
Metallic Pastes for Filling Teeth, 210
McCabe, J. D., D. D. S., Address before the Virginia Society of Surgeon Dentists, on the Importance of thorough Dental Education, . . . 169
McCabe, J. C., Dr., Letter from, 88
Mineral Paste, for Filling Teeth, 256

N

Notice, . . . . . . 72
Neuralgia from Sympathy with a Diseased Tooth, . 120
C. L. Norton, Dentist, of New York, Letter from, . . 266

O

Opening Address, by C. A. Harris, M. D., . . . . 3
Operative and Mechanical Dentistry, Contributions to, by W. H. Elliott, . . . . 45–162
Operation for Cataract, . 128
Observations on the Extraction of Teeth, by J. Chitty Clendon, 128

PAGE.
Our Work, . . . . 133
Onondaga County Medical Society, Report of the, on Mineral Paste, by A. Wescott, M. D., . . . . . 175
Obituary of H. H. Hayden, M. D., . . . . . 133

P

Potatoes, a Preventive and Cure for Scurvy, . . . . 66
Persons, the way some become Dentists, . . . . . 136

Q

Quackery, the last Importation of transatlantic, &c., . 71
Quackery, Illustrations and Sketches of Medical, . 121
Quackery, . . . . . 289

R

Robinson, Dr. B. R., . . 71
Report of the Onondaga Co. Medical Society on Mineral Paste, by A. Wescott, M. D. 175
Rogers, M., M. D., Address delivered before the Cincinnati Association of Dental Surgeons, . . . . . 258

S

Shepherd, S. M., D. D. S., on Alveolar Exostosis, . . 53
Superior Denture, Irregularity of the, by Ed. Maynard, M. D., 55
Staphyloraphy, . . . 60
Stammering . . . . 61
Science of Meteorology . . 62
Surgery, Dental, . . . 66
Science of the Human Body, by W. R. Handy, M. D., . 77
Swallowing a five franc piece, 124
Secret Remedies in France, 125
Salivary Calculus, . . . 141
Somerby, Dr. R., his Concentrated Blow-pipe, . . . 218
Subscribers, to our, . . . 300

PAGE.

T

Tooth, cure of Abscess of, . 58
Teeth, Artificial, . . . 72
" Caries of the, by Dr. Wescott, . . . . . 73
Teeth, Morbid Sympathies existing between the, and other parts of the Body, by Professor Bond, . . . 74
Teeth, Tartar on the, . . 118
" Mineral Paste for filling, 256
Thackston, W. W. H., D. D. S. on Extracting Forceps, . 166
and on Morbid Effects of diseased Teeth, &c., &c., . 230
Transactions of the Virginia Society of Surgeon Dentists, 115
Tonsils, Enlarged and Uvula Elongated, . . . 61
Tartar or Calculus of the Mouth, translated from the French of Delabarre, by C. A. Harris, M. D., . . . . . . 268
Teeth, Filing the, . . 297
Trachea, Rupture of the . 298

PAGE.

U

Uvula, Elongated, and Enlarged Tonsils, . . . . . 61
Ulceration of the Gums, &c., by Prof. C. A. Harris, . . 93

V

Virginia Society of Dental Surgeons, Address before the, by J. D. McCabe, D. D. S. . 99
Valedictory Address, by Prof. T. E. Bond, M. D., . . . . 221

W

Wescott, A., M. D., Dissertation on Dental Caries, . 31–73
Wescott, Report of the Onondaga County Medical Society on Mineral Paste, . . . 175
Wells, Artisian, . . . . 64
Writers, Medical, good Practitioners, . . . . . 206

The Treasurer has taken much pains in making out accounts against those who were indebted to the Society for the Journal, and annual dues. He takes occasion to thank those who have promptly responded to his call, and would respectfully urge such as have not remitted the amount due, to do so at once, as the Society is in debt, and he has no other source to look to for relief. The Treasurer may have committed some mistakes in making out bills, owing to the confusion of the books which came to his hands, but he will gladly correct all errors.

☞ Agents are respectfully requested to collect and transmit, as speedily as possible, to the undersigned, such moneys as are due for the Journal. L. MACKALL, *Treas.*

### RECEIPTS.

$100 have been received from John Lees, Philadelphia agent, at sundry times, but he has not furnished the names of the persons from whom he had made collections; when furnished, they will be published.

Blandin & Avery, ..........Columbia, S. C.

Eleazar Gidney,............Manchester, Eng.

Drs. Goddard and Ferguson,Louisville, Ky.

James Taylor, M. D.. .....Cincinnati, Ohio.

